# Morin Attenuates Hyperglycemia and Metabolic Dysregulation in Ovariectomized Diabetic Mouse Model

**DOI:** 10.3390/medsci14030371

**Published:** 2026-07-03

**Authors:** Josué Vidal Espinosa-Juárez, Viridiana Orantes-Sánchez, Joaquín Gómez-Morga, Citlaly Natali de la Torre-Sosa, Alfredo Briones-Aranda, Osmar Antonio Jaramillo-Morales, Josselin Carolina Corzo-Gómez, Refugio Cruz-Trujillo, Raúl Cruz-Cadena, Raquel Gómez Pliego

**Affiliations:** 1 Escuela de Ciencias Químicas, Universidad Autónoma de Chiapas (UNACH), Carretera Panamericana Ocozocoautla-Cintalapa Km. 2.5, Ocozocoautla de Espinosa 29140, Chiapas, Mexico; josue.espinosa@unach.mx (J.V.E.-J.); an180009@unach.mx (V.O.-S.); an191018@unach.mx (J.G.-M.); citlaly.torre@unach.mx (C.N.d.l.T.-S.); josselin.corzo@unach.mx (J.C.C.-G.); refugio.cruz@unach.mx (R.C.-T.); cruz.raul@unach.mx (R.C.-C.); 2 Facultad de Medicina Humana Campus II, Universidad Autónoma de Chiapas (UNACH), Décima Sur Esquina Calle Central S/N, Tuxtla Gutiérrez 29050, Chiapas, Mexico; 3 Departamento de Enfermería y Obstetricia, División de Ciencias de la Vida, Campus Irapuato-Salamanca, Universidad de Guanajuato, Ex Hacienda el Copal, Km. 9 Carretera Irapuato-Silao, A.P. 311, Irapuato 36500, Guanajuato, Mexico; oa.jaramillo@ugto.mx; 4 Departamento de Químicos Farmacobiólogos, Universidad Pablo Guardado Chávez (UPGCH), Libramiento Norte Oriente No. 3450, Tuxtla Gutiérrez 29040, Chiapas, Mexico; 5 Sección de Ciencias de la Salud Humana, Departamento de Ciencias Biológicas, Facultad de Estudios Superiores Cuautitlán, Universidad Nacional Autónoma de México, Cuautitlán Izcalli 54740, Estado de México, Mexico

**Keywords:** diabetes, flavonoid, hypoestrogenism, morin

## Abstract

**Background/Objectives:** Estrogen deficiency is associated with metabolic disturbances and impaired glucose homeostasis. Morin, a natural flavonol, has shown promising hypoglycemic and antioxidant properties, but its effects under hypoestrogenic diabetic conditions remain poorly understood. The aim of this study was to evaluate the effects of morin on body weight, fasting blood glucose, glucose tolerance, and selected serum biochemical markers in an experimental model of diabetes under estrogen-deficient conditions (ovariectomized diabetic female mice). **Methods:** Female CD1 mice underwent sham surgery or ovariectomy (OVX), and each surgical condition was further divided into non-diabetic and diabetic subgroups treated with vehicle, glibenclamide (10 mg/kg), or morin (30 mg/kg). Body weight and fasting blood glucose were monitored over a 15-day treatment period. Oral glucose tolerance was assessed on day 15, and serum biochemical markers, including glucose, cholesterol, triglycerides, uric acid, blood urea nitrogen, creatinine, ALT, and AST, were measured thereafter. **Results:** Ovariectomy aggravated diabetes-associated hyperglycemia, impaired glucose tolerance, and triglyceride elevation. Morin treatment reduced fasting blood glucose and improved glucose tolerance in diabetic mice, including ovariectomized animals. Morin also attenuated the increase in serum triglycerides and blood urea nitrogen in ovariectomized diabetic mice, although it did not significantly improve cholesterol, uric acid, creatinine, ALT, or AST levels. Compared with glibenclamide, morin showed relevant glucose-lowering activity but had a more limited effect on the overall biochemical profile. **Conclusions:** These findings suggest that morin may partially improve glycemic control and selected metabolic alterations in experimental diabetes associated with estrogen deficiency. Further studies are required to clarify its mechanisms of action, long-term efficacy, and translational relevance.

## 1. Introduction

Diabetes mellitus (DM) is a metabolic disorder characterized by chronic hyperglycemia; alterations in carbohydrate, lipid, and protein metabolism; and progressive deterioration of pancreatic β-cells [1]. Due to its high global prevalence and chronic complications, DM remains one of the leading causes of premature morbidity and mortality in most developed and developing countries [2]. Recent evidence indicates that postmenopausal women are at increased risk of developing type 2 DM, highlighting the relevance of estrogen deficiency in metabolic dysfunction [3].

Estrogen deficiency is clinically relevant to glucose and lipid dysregulation because estrogens contribute to the regulation of insulin sensitivity, hepatic glucose production, adipose tissue distribution, lipid handling, mitochondrial function, and oxidative stress [4,5,6]. Accordingly, the menopausal transition is associated with changes in body composition, increased adiposity, impaired glucose homeostasis, altered lipid metabolism, and greater susceptibility to metabolic deterioration [4,7,8]. Therefore, experimental approaches that combine estrogen deprivation with diabetic metabolic stress may be useful to evaluate compounds capable of modulating systemic metabolic alterations under conditions that partially resemble hypoestrogenic metabolic vulnerability.

Animal models have been instrumental in studying the interaction between hormonal deficiency and diabetes. Ovariectomy (OVX) is widely used to reproduce selected features of estrogen deprivation in rodents [9,10,11]. In the present study, alloxan was selected because it produces a reproducible hyperglycemic state through reactive oxygen species-mediated pancreatic β-cell injury and is commonly used to evaluate the glucose-lowering activity of natural compounds [12,13]. Although streptozotocin and diet-induced models represent alternative approaches for studying experimental diabetes and metabolic dysfunction, the OVX/alloxan model was chosen as a controlled proof-of-concept strategy to evaluate short-term systemic metabolic responses under combined estrogen-deficient and diabetic conditions, while avoiding additional variability related to prolonged dietary manipulation [13,14]. However, alloxan-induced diabetes primarily reflects β-cell toxicity and insulin-deficient hyperglycemia rather than the insulin resistance-dominant pathophysiology of human type 2 diabetes [13,14]. Thus, although the OVX/alloxan model does not fully reproduce the complexity of clinical metabolic disease, it provides a controlled experimental approach to evaluate short-term systemic metabolic responses under conditions combining ovariectomy and chemically induced diabetes.

Medicinal plants and their bioactive compounds have been widely explored as potential sources of therapeutic agents for diabetes management [15]. Morin (MOR) is a naturally occurring flavonol found in several edible and medicinal plants, including species of the Moraceae family, and has been associated with antioxidant, anti-inflammatory, hypoglycemic, and lipid-modulating properties [16,17,18]. In experimental models of diabetes and metabolic dysfunction, MOR has been reported to improve glucose handling, modulate lipid metabolism, reduce oxidative stress, and attenuate pancreatic, hepatic, or renal alterations [17,19,20]. MOR was selected for the present study because its reported glucose-lowering and antioxidant-related properties make it a relevant candidate for evaluating metabolic modulation under hypoestrogenic diabetic conditions. However, its effects in the combined context of estrogen deficiency and experimental diabetes remain poorly understood.

Glibenclamide (GLI), a second-generation sulfonylurea, was used as the reference drug because it lowers blood glucose mainly by stimulating insulin secretion through the inhibition of ATP-sensitive potassium channels in pancreatic β-cells [21,22]. Its inclusion allowed comparison of MOR with an established glucose-lowering agent under the same OVX/alloxan-induced diabetic conditions.

Based on this background, we hypothesized that MOR treatment would attenuate hyperglycemia, impaired glucose tolerance, and selected biochemical alterations in ovariectomized diabetic female mice. The novelty of this study lies in evaluating the short-term metabolic effects of MOR in a combined model of estrogen deficiency and alloxan-induced diabetes, rather than in diabetes alone. Therefore, the objective of this study was to evaluate the effect of MOR on body weight, fasting glucose, glucose tolerance, and selected serum biochemical markers in ovariectomized female mice with alloxan-induced diabetes.

## 2. Materials and Methods

### 2.1. Animals

Forty-eight adults female CD1 mice (7 weeks old, 25–30 g) were obtained from the Animal Facility of the Faculty of Human Medicine, Universidad Autónoma de Chiapas, Mexico. The animals were acclimatized to laboratory environmental conditions for one week and housed in polycarbonate cages (42 × 30 × 27 cm) under controlled conditions (22 ± 2 °C; 12 h light/dark cycle). They had free access to standard rodent pellet food and water ad libitum throughout the experimental period. All experimental procedures complied with the Mexican Official Standard NOM-062-ZOO-1999 [23] for the use and care of laboratory animals and were approved by the Research Committee of the Universidad Autónoma de Chiapas under protocol number 03/ECQ/RPR/066/22. Animals were monitored daily throughout the acclimatization, postoperative, diabetic induction, and treatment periods. No unexpected adverse events or deaths occurred during the study. All procedures were performed with the aim of minimizing pain, suffering, and distress.

### 2.2. Drugs and Treatments

Morin hydrate, alloxan, and carboxymethylcellulose (CMC) were purchased from Sigma-Aldrich (St. Louis, MO, USA). Glibenclamide (GLI) was obtained from Laboratorios PiSA (Guadalajara, Jalisco, Mexico), and sodium pentobarbital was obtained from Laboratorios Aranda (Mexico City, Mexico).

### 2.3. Pharmacological Regimens

MOR and GLI were suspended in 0.5% CMC in sterile saline immediately before use and administered by intraperitoneal injection (i.p.). The MOR dose (30 mg/kg) used in this study was selected based on previous reports showing that morin at this dose range exerts glucose-lowering, antioxidant, and metabolic regulatory effects in experimental models of diabetes and metabolic dysfunction [19,24]. The doses of the reference drug GLI (10 mg/kg, i.p.) [21] and the anesthetic pentobarbital (20 mg/kg, i.p.) [25] were also based on earlier studies. Control animals received intraperitoneal injections of 0.5% CMC solution as vehicle.

The intraperitoneal route was selected to ensure controlled systemic exposure and reduce variability associated with gastrointestinal absorption during this proof-of-concept study. This approach allowed a consistent comparison between MOR and GLI under OVX/alloxan-induced diabetic conditions. Control animals received 0.5% carboxymethylcellulose solution (1 mL/kg, i.p.) as a vehicle. Alloxan was freshly dissolved in saline and administered intraperitoneally at 200 mg/kg fifteen days after OVX or sham surgery, following a 12 h fast [13,26].

### 2.4. Experimental Design

**Figure 1:**
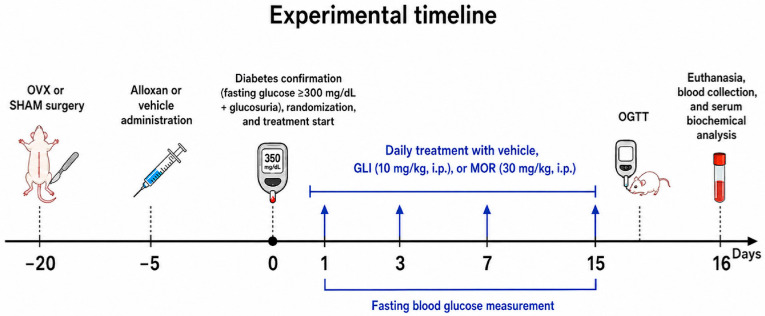
Days are shown relative to treatment start. Day 0 was defined as diabetes confirmation, randomization, and treatment initiation. OVX/SHAM surgery was performed on day −20; alloxan/vehicle administration on day −5; fasting blood glucose measurements on days 1, 3, 7, and 15; OGTT on day 15; and blood collection with serum biochemical analysis on day 16.

The diabetes induction protocol was selected to evaluate short-term systemic metabolic responses under combined estrogen-deficient and diabetic conditions. Alloxan was used because it induces reproducible hyperglycemia through reactive oxygen species-mediated pancreatic β-cell injury and has been widely used to assess the glucose-lowering activity of natural compounds [12,13]. The 15-day interval between OVX or sham surgery and alloxan administration was selected to allow postoperative recovery and the establishment of early estrogen deficiency-related metabolic changes before the induction of diabetic stress [9,10,27]. Five days after alloxan or vehicle administration, fasting blood glucose levels were measured (Figure 1).

After the completion of the surgical and diabetic induction procedures, eligible mice were randomly allocated to the corresponding experimental groups using a manual randomization procedure, without computer-generated allocation sequences. Group sizes were kept balanced across the experimental conditions. Mice were distributed into eight experimental groups with six animals per group, and the sample size was established based on previous studies using similar experimental models and biochemical endpoints, as well as ethical considerations to minimize animal use while maintaining sufficient group sizes for statistical comparison.

Biochemical parameters were evaluated using coded serum samples. The investigator performing the biochemical determinations was unaware of the experimental group allocation at the time of analysis. Blinding was maintained until completion of the biochemical measurements and data recording.

The first four groups consisted of sham-operated (SHAM) mice (Figure 1). Group 1 served as the control and received the vehicle (SHAM + VEH). Group 2 included SHAM diabetic mice treated with the vehicle (SHAM + DIAB + VEH). Groups 3 and 4 consisted of SHAM diabetic mice treated once daily for fifteen days with MOR (SHAM + DIAB + MOR) or GLI (SHAM + DIAB + GLI), respectively. The remaining four groups of OVX mice received equivalent pharmacological treatments: group 5 (OVX + VEH), group 6 (OVX + DIAB + VEH), group 7 (OVX + DIAB + MOR), and group 8 (OVX + DIAB + GLI).

Body weight and fasting blood glucose were recorded before treatment initiation and throughout the 15-day experimental period. On day 16, the animals were euthanized, and serum samples were collected for biochemical analysis.

### 2.5. Experimental Procedures

#### 2.5.1. Ovariectomy

Mice were anesthetized with pentobarbital. The ovaries were located and removed bilaterally. The peritoneum and muscle layers were closed with absorbable sutures (Ethicon chromic sutures 4/0, Johnson & Johnson Medical Devices, Irvine, CA, USA), and the skin was closed with nonabsorbable nylon sutures (Ethicon mononylon sutures 4/0, Johnson & Johnson Medical Devices, USA), which spontaneously detached 2–3 days after surgery [25]. Sham-operated animals underwent the same procedure without ovary removal. Successful ovariectomy was inferred from the surgical procedure and the postoperative recovery period, which are consistent with established protocols to induce hypoestrogenic conditions in rodents. However, uterine weight and circulating estradiol levels were not measured in this study [9].

#### 2.5.2. Induction of Experimental Diabetes

Fifteen days after OVX or sham surgery, and following a 12 h fast, mice received a single intraperitoneal injection of freshly prepared alloxan (200 mg/kg in saline). After five days (Figure 1), blood glucose was measured, and animals with fasting glucose levels ≥ 300 mg/dL accompanied by glucosuria were considered diabetic and included in the study.

#### 2.5.3. Blood Glucose Measurement

Mice were fasted for 12 h before testing. Blood glucose levels were determined 1 h after drug administration on days 1, 3, 7, and 15 of treatment (Figure 1). Blood samples were collected from the tail vein (saphenous puncture), and glucose concentrations were measured using an Accu-Chek^®^ Performa glucometer (Roche Diagnostics, Mannheim, Germany).

#### 2.5.4. Oral Glucose Tolerance Test (OGTT)

On day 15 after treatment (Figure 1), mice were fasted for 12 h and administered glucose orally at a dose of 2 g/kg. Blood glucose was measured at baseline (0) and at 30, 60, 90, and 120 min after glucose administration.

#### 2.5.5. Biochemical Analysis

On day 16, mice were euthanized by decapitation, and blood samples were collected. Serum was obtained by centrifugation at 4500 rpm for 15 min and analyzed for cholesterol, triglycerides, uric acid, blood urea nitrogen (BUN), creatinine alanine aminotransferase (ALT), and aspartate aminotransferase (AST) using the VITROS^®^ 4600 Chemistry System (Ortho Clinical Diagnostics, Raritan, NJ, USA).

### 2.6. Statistical Analysis

Data are expressed as mean ± standard error of the mean (SEM). Normality was evaluated using the Shapiro–Wilk test. Longitudinal data, including body weight, fasting blood glucose over the treatment period, and OGTT curves, were analyzed using two-way repeated-measures analysis of variance (ANOVA), with time as the within-subject factor and experimental group/treatment as the between-subject factor. When appropriate, significant main effects or interactions were followed by post hoc multiple comparisons adjusted using Tukey’s test. Serum biochemical parameters measured at a single time point were analyzed using two-way ANOVA followed by Tukey’s post hoc test. Differences were considered statistically significant at *p* < 0.05. All analyses were conducted using GraphPad Prism 6.0 software (GraphPad Software, San Diego, CA, USA).

## 3. Results

### 3.1. Body Weight

**Figure 2:**
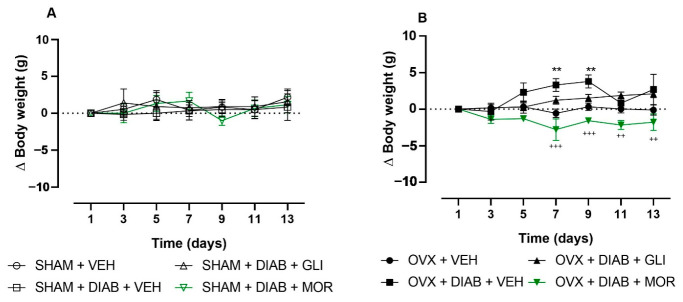
Body weight dynamics during treatment: (**A**) SHAM subgroups; (**B**) OVX subgroups. Groups: SHAM + VEH, SHAM + DIAB + VEH, SHAM + DIAB + GLI (glibenclamide 10 mg/kg, i.p.), SHAM + DIAB + MOR (morin 30 mg/kg, i.p.), OVX + VEH, OVX + DIAB + VEH, OVX + DIAB + GLI (10 mg/kg, i.p.), and OVX + DIAB + MOR (30 mg/kg, i.p.). Data are expressed as mean ± SEM (*n* = 6/group). ** *p* < 0.01 vs. OVX + DIAB + VEH; ^++^ *p* < 0.01, ^+++^ *p* < 0.001 vs. OVX + VEH.

Figure 2 shows body-weight changes during the treatment period in SHAM-operated (Figure 2A) and OVX (Figure 2B) mice. In SHAM-operated diabetic mice, no significant differences in body weight were observed among vehicle-, GLI-, and MOR-treated groups. In OVX mice, the OVX + DIAB + VEH group showed a progressive increase in body weight compared with the OVX + VEH group (*p* < 0.01, *p* < 0.001). This increase was attenuated in the OVX + DIAB + GLI and OVX + DIAB + MOR groups, which showed significantly lower body-weight values compared with OVX + DIAB + VEH mice (*p* < 0.01, *p* < 0.001) during the treatment period (Figure 2B). The most evident body-weight differences were observed in the OVX diabetic condition, whereas SHAM diabetic mice showed no marked treatment-related changes.

### 3.2. Blood Glucose

**Figure 3:**
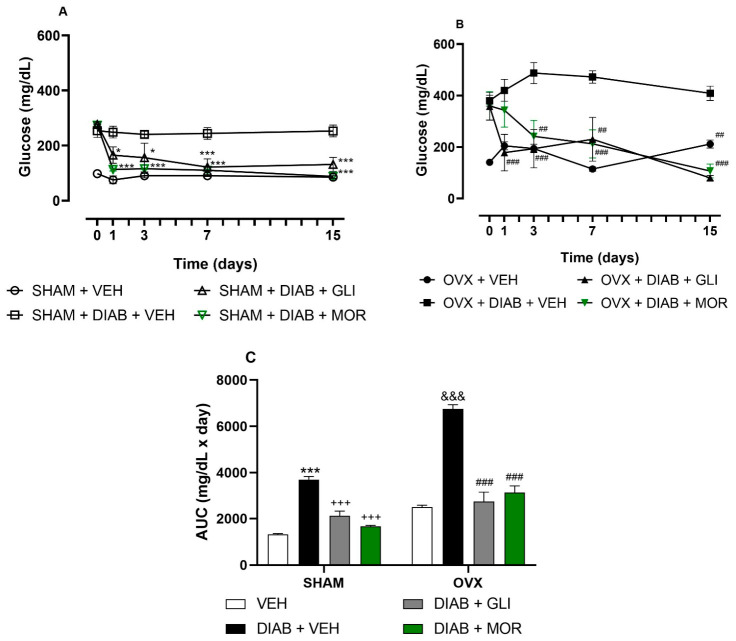
Fasting blood glucose during the 15-day treatment period. (**A**) SHAM-operated groups. (**B**) OVX groups. (**C**) Area under the curve (AUC). Data are expressed as mean ± SEM (*n* = 6/group). * *p* < 0.05 and *** *p* < 0.001 vs. SHAM + VEH; ^+++^ *p* < 0.001 vs. SHAM + DIAB + VEH; ^##^ *p* < 0.01, ^###^ *p* < 0.001 vs. OVX + DIAB + VEH; ^&&&^ *p* < 0.001 vs. OVX + VEH.

Figure 3 shows the longitudinal changes in fasting blood glucose during the 15-day treatment period. Vehicle-treated diabetic mice showed higher glucose levels than their corresponding non-diabetic controls in both SHAM and OVX conditions throughout the study. Among non-diabetic animals, OVX + VEH mice showed higher glucose values than SHAM + VEH mice during the initial days of follow-up.

In SHAM diabetic mice (Figure 3A), both GLI and MOR reduced fasting blood glucose compared with SHAM + DIAB + VEH. GLI produced an earlier decrease, with significant differences from day 3 onward (*p* < 0.001), whereas MOR showed a gradual reduction that reached statistical significance from day 7 onward (*p* < 0.01 to *p* < 0.001). In OVX diabetic mice (Figure 3B), both GLI and MOR also reduced fasting blood glucose compared with OVX + DIAB + VEH, with significant reductions observed from day 3 for GLI and from day 7 for MOR (*p* < 0.01 to *p* < 0.001). The AUC analysis (Figure 3C) showed significant effects of surgical condition, treatment, and their interaction on total glycemic exposure. OVX + DIAB + VEH mice showed higher AUC values than SHAM + DIAB + VEH mice (*p* < 0.001). Treatment with GLI or MOR reduced AUC values in both SHAM and OVX diabetic mice compared with their respective vehicle-treated diabetic groups (*p* < 0.001).

### 3.3. Oral Glucose Tolerance Test (OGTT)

**Figure 4:**
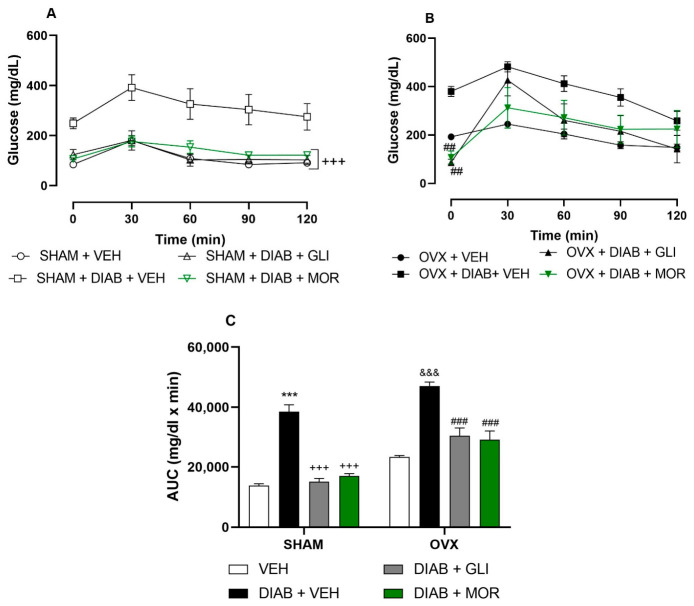
Oral glucose tolerance test. Mice received an oral glucose load (2 g/kg), and blood glucose was measured at 0, 30, 60, 90, and 120 min. (**A**) SHAM-operated groups, (**B**) OVX groups, and (**C**) AUC analysis of the OGTT. Data are expressed as mean ± SEM (*n* = 6/group). *** *p* < 0.001 vs. SHAM + VEH; ^+++^ *p* < 0.001 vs. SHAM + DIAB + VEH; ^&&&^ *p* < 0.001 vs. OVX + VEH; ^##^ *p* < 0.01, ^###^ *p* < 0.001 vs. OVX + DIAB + VEH.

Figure 4 shows the glucose response during the OGTT performed on day 15. In SHAM diabetic mice (Figure 4A), GLI and MOR reduced post-load glucose levels compared with SHAM + DIAB + VEH, with significant differences observed from 30 min onward (*p* < 0.001). In OVX diabetic mice (Figure 4B), both treatments also reduced glucose levels compared with OVX + DIAB + VEH, although glucose values remained higher during the first part of the curve than those observed in treated SHAM diabetic mice. The AUC analysis (Figure 4C) showed significant effects of surgical condition (*p* < 0.001) and treatment (*p* < 0.001), whereas the interaction between these factors was not significant (*p* > 0.05). Vehicle-treated diabetic mice showed higher AUC values than their respective non-diabetic controls (*p* < 0.001). Treatment with GLI or MOR reduced total glycemic exposure in both SHAM and OVX diabetic mice compared with their corresponding vehicle-treated diabetic groups (*p* < 0.001).

Direct comparisons of OGTT AUC values across surgical conditions showed higher total glycemic exposure in OVX + DIAB + VEH mice than in SHAM + DIAB + VEH mice (*p* < 0.05). In MOR-treated diabetic mice, OGTT AUC values were higher in OVX + DIAB + MOR than in SHAM + DIAB + MOR mice (*p* < 0.001). However, the nonsignificant surgical condition × treatment interaction indicated that the glucose tolerance-improving effect of MOR was not statistically different between SHAM and OVX diabetic mice under the present experimental conditions. Therefore, although MOR improved glucose tolerance in both SHAM and OVX diabetic mice, the OVX condition was associated with higher residual glycemic exposure after treatment.

### 3.4. Biochemical Parameters

**Figure 5:**
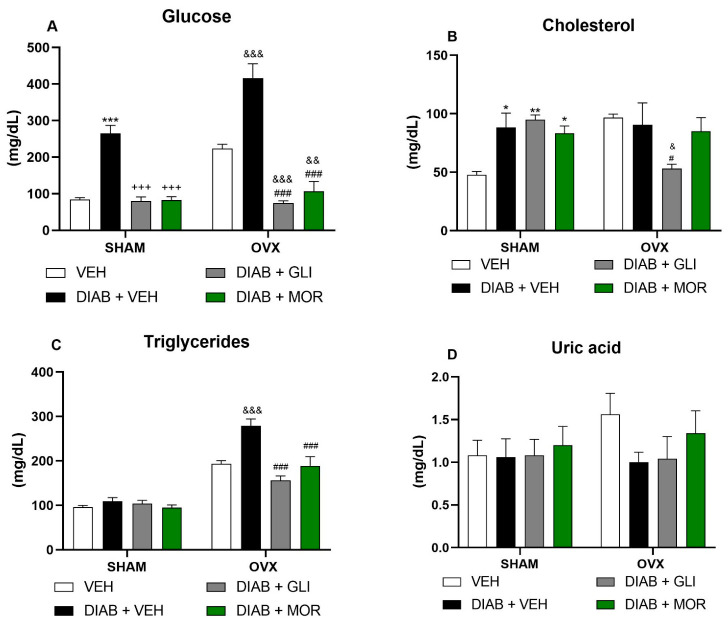
Effects of MOR and GLI on glycemic and metabolic serum biochemical parameters. (**A**) Glucose, (**B**) cholesterol, (**C**) triglycerides, and (**D**) uric acid levels measured after 15 days of treatment in SHAM-operated and OVX female mice with or without alloxan-induced diabetes. GLI: glibenclamide 10 mg/kg, i.p.; MOR: morin 30 mg/kg, i.p.; VEH: vehicle. Data are expressed as mean ± SEM (*n* = 6/group). * *p* < 0.05, ** *p* < 0.01, *** *p* < 0.001 vs. SHAM + VEH group; ^+++^ *p* < 0.001 vs. SHAM + DIAB + VEH; ^&^ *p* < 0.05, ^&&^ *p* < 0.01 and ^&&&^ *p* < 0.001 vs. OVX + VEH group; ^#^ *p* < 0.05 and ^###^ *p* < 0.001 vs. OVX+ DIAB + VEH group.

After 15 days of treatment, endpoint serum biochemical parameters were analyzed. Figure 5 shows glycemic and metabolic serum markers. Day-15 glucose levels (Figure 5A) were higher in vehicle-treated diabetic mice than in their corresponding non-diabetic controls. Treatment with GLI or MOR reduced glucose levels in both SHAM and OVX diabetic mice compared with their respective vehicle-treated diabetic groups (*p* < 0.001). Cholesterol levels (Figure 5B) were increased in diabetic mice compared with non-diabetic controls; this increase persisted in MOR-treated diabetic mice, whereas lower cholesterol values were observed in the GLI-treated OVX diabetic group. Triglyceride levels (Figure 5C) were higher in OVX mice, particularly in the OVX + DIAB + VEH group. Treatment with GLI or MOR reduced triglyceride levels in OVX diabetic mice compared with OVX + DIAB + VEH mice (*p* < 0.001). No significant differences were detected in uric acid levels among groups (Figure 5D).

**Figure 6:**
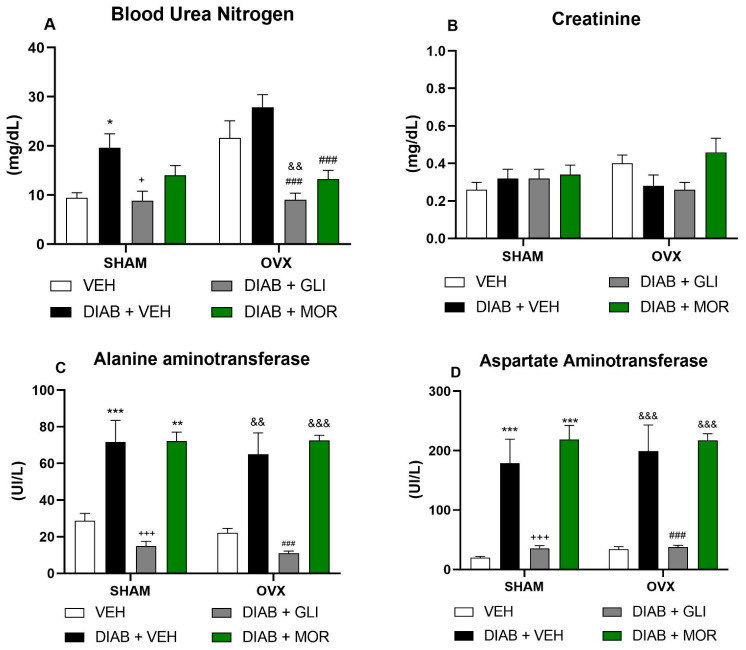
Effects of MOR and GLI on renal- and hepatic-function-related serum biochemical markers. (**A**) Blood urea nitrogen (BUN), (**B**) creatinine, (**C**) alanine aminotransferase (ALT), and (**D**) aspartate aminotransferase (AST) levels measured after 15 days of treatment in SHAM-operated and OVX female mice with or without alloxan-induced diabetes. GLI: glibenclamide 10 mg/kg, i.p.; MOR: morin 30 mg/kg, i.p.; VEH: vehicle. Data are expressed as mean ± SEM (*n* = 6/group). * *p* < 0.05, ** *p* < 0.01, *** *p* < 0.001 vs. SHAM + VEH group; ^+^ *p* < 0.05 and ^+++^ *p* < 0.001 vs. SHAM + DIAB + VEH; ^&&^ *p* < 0.01 and ^&&&^ *p* < 0.001 vs. OVX + VEH group; ^###^ *p* < 0.001 vs. OVX + DIAB + VEH group.

Figure 6 shows renal- and hepatic-function-related serum biochemical markers. BUN levels (Figure 6A) were increased in vehicle-treated diabetic mice, with higher values observed in the OVX + DIAB + VEH group. Treatment with GLI or MOR reduced BUN levels compared with OVX + DIAB + VEH mice (*p* < 0.001). No significant differences were detected in creatinine levels among groups (Figure 6B). ALT and AST levels (Figure 6C,D) were increased in diabetic groups compared with non-diabetic controls. GLI reduced both enzymes in SHAM and OVX diabetic mice (*p* < 0.001), whereas MOR did not significantly reduce ALT or AST levels.

## 4. Discussion

In this study, OVX alone did not induce apparent changes in body weight in non-diabetic mice. This finding is consistent with previous studies conducted at similar postoperative time points [10,27] and contrasts with other reports [28]. This apparent discrepancy may be related to the gradual weight gain observed in OVX mice, which tends to become evident at later time points [11,27].

However, when OVX was combined with diabetes induction, there was a marked increase in body weight and hyperglycemia, as well as impaired glucose tolerance, compared with non-diabetic SHAM or OVX groups. These results are consistent with reports showing marked metabolic disruption in streptozotocin-induced diabetic mice [29] and align with findings showing that OVX, when combined with a high-fat, high-sucrose diet, accelerates the onset of diabetes. In contrast, non-OVX female mice exposed only to the hypercaloric diet developed diabetes later [30]. The exacerbated metabolic phenotype observed in OVX + DIAB mice may reflect the combined impact of alloxan-induced pancreatic β-cell injury and estrogen deficiency-associated metabolic vulnerability. Alloxan primarily induces insulin-deficient hyperglycemia through reactive oxygen species-mediated β-cell damage [13], whereas estrogen deficiency has been associated with impaired insulin sensitivity, altered hepatic glucose regulation, adipose tissue remodeling, lipid dysregulation, and oxidative stress-related pathways [6,8,27]. Therefore, the OVX + DIAB condition may represent a compounded metabolic challenge involving impaired insulin secretion together with estrogen deficiency-related alterations in insulin action and lipid handling. This dual condition may help explain the more pronounced hyperglycemia, impaired glucose tolerance, triglyceride elevation, and BUN changes observed in OVX diabetic mice. However, because insulin levels, pancreatic β-cell integrity, insulin signaling, renal tissue analyses, and oxidative stress markers were not assessed, this interpretation should be considered pathophysiological and hypothesis-generating.

The increase in body weight observed in OVX + DIAB + VEH mice should be interpreted cautiously. Although ovariectomy and experimental diabetes may influence energy balance and systemic metabolic regulation, the present data do not allow for the determination of the physiological basis of this body-weight change. Parameters such as food intake, water intake, urine output, body composition, adipose tissue mass, and lean mass were not measured. Therefore, the observed increase in Δ body weight should not be directly attributed to increased adiposity, fluid retention, or improved metabolic regulation. Similarly, the lower Δ body weight values observed in MOR-treated mice should be interpreted as an attenuation of body-weight gain under the present experimental conditions, rather than as definitive evidence of improved body-weight-related metabolic regulation. Further studies using metabolic cage analyses, body-composition measurements, and tissue-level assessments are required to clarify the mechanisms underlying these changes [6,8,27,31].

An important finding of the present study is that MOR progressively reduced blood glucose levels and improved glucose tolerance in diabetic mice, including OVX diabetic animals. However, OVX + DIAB + MOR mice maintained higher residual glycemic exposure than SHAM + DIAB + MOR mice, suggesting that MOR retained glucose tolerance-improving activity under estrogen-deficient diabetic conditions but did not fully normalize the aggravated glycemic response associated with OVX. These results are consistent with previous reports in diabetic male rats showing antihyperglycemic effects of MOR and GLI [18,21,24]. Previous studies have reported that MOR can modulate glycogenesis, gluconeogenesis, Akt and insulin receptor phosphorylation, and PTP1B activity in experimental systems [20,32]. These mechanisms may help contextualize the glucose-lowering profile observed in the present study; however, insulin signaling and hepatic glucose metabolism were not directly assessed here. Similarly, although estrogen deficiency and MOR have both been linked to oxidative stress-related pathways [4,8,33], these pathways were not directly evaluated in the present study.

The biochemical profile further supports the interaction between estrogen deficiency, diabetes, and systemic metabolic stress. Although uric acid and creatinine remained unchanged, and MOR did not clearly improve cholesterol levels, ALT and AST levels were increased in diabetic groups, indicating changes in serum hepatic injury-related markers under the present experimental conditions. However, MOR did not significantly reduce ALT or AST levels, whereas GLI reduced both enzymes in SHAM and OVX diabetic mice. Therefore, the present data do not support a direct hepatic protective effect of MOR in this model. Although previous studies have reported antioxidant and cytoprotective effects of MOR in other experimental settings [18,32,34], additional analyses, including liver histology, hepatic oxidative stress markers, inflammatory mediators, and metabolic enzyme expression, are required to determine whether MOR exerts tissue-level hepatic effects under hypoestrogenic diabetic conditions.

It is also important to consider the partial nature of the biochemical response to MOR. Although MOR reduced glucose, triglycerides, and BUN in OVX diabetic mice, it did not significantly improve cholesterol, creatinine, ALT, or AST levels. The absence of a clear effect on cholesterol suggests that the lipid-modulating action of MOR was mainly reflected in triglyceride levels under the present experimental conditions. Similarly, creatinine remained unchanged among groups, indicating that the reduction in BUN should be interpreted cautiously and not as evidence of a broad improvement in renal function. Regarding hepatic markers, the lack of significant reductions in ALT and AST indicates that MOR did not attenuate serum hepatic injury-related markers in this model. Therefore, the overall biochemical profile suggests a selective metabolic effect of MOR rather than a generalized renal or hepatic protective effect.

The present study has several limitations that should be considered. Serum 17β-estradiol and uterine weight were not evaluated; however, the ovariectomy procedure and postsurgical interval employed are consistent with hypoestrogenic conditions reported in previous OVX-based rodent models [9,10,25]. Nevertheless, the absence of direct hormonal confirmation should be acknowledged as a limitation. Although the group size was selected considering previous reports and ethical principles aimed at reducing animal use, the sample size was small (*n* = 6 per group), which may limit the statistical power to detect subtle treatment effects or weaker interactions among surgical condition, diabetes, and treatment [35]. In addition, only one dose of MOR was evaluated, preventing assessment of dose–response relationships. MOR was administered intraperitoneally to ensure controlled systemic exposure in this proof-of-concept study; however, this route does not reproduce gastrointestinal absorption or first-pass processes associated with oral administration, limiting direct translational extrapolation [36]. Similarly, the 15-day observation period allowed evaluation of short-term glycemic and biochemical responses but does not support conclusions regarding sustained efficacy, long-term safety, or durability of response.

The OVX/alloxan-induced diabetes model reproduces selected aspects of experimental diabetes under estrogen-deficient conditions; however, it does not fully reflect the multifactorial pathophysiology of human metabolic disease. In particular, alloxan induces diabetes primarily through pancreatic β-cell injury and oxidative stress; therefore, this model mainly reflects β-cell toxicity rather than an insulin resistance-driven diabetic phenotype [13]. In addition, the biochemical analysis was restricted to systemic serum markers, and insulin levels, HOMA-IR, insulin tolerance testing, oxidative stress markers, inflammatory mediators, and tissue-level analyses were not performed. Therefore, the present data do not allow us to determine whether the effects of MOR were related to improved insulin sensitivity, preserved pancreatic β-cell function, reduced hepatic glucose production, antioxidant activity, or other indirect metabolic effects. Although MOR has been associated with antioxidant properties [18,33], oxidative stress-related markers were not measured; thus, any antioxidant contribution of MOR in this model remains speculative.

Future studies should prioritize three mechanistic areas directly related to the present findings: insulin and β-cell function, oxidative stress-related pathways, and lipid or renal metabolic alterations under hypoestrogenic diabetic conditions. Complementary models of insulin resistance, diet-induced obesity, or postmenopausal metabolic dysfunction will also be useful to confirm the metabolic effects of MOR and improve their translational relevance. In addition, oral efficacy, bioavailability, dose–response relationships, and long-term safety should be evaluated before considering translational applications of MOR under hypoestrogenic diabetic conditions.

## 5. Conclusions

The present study provides preclinical evidence that MOR exerts a short-term glucose-lowering effect and partially improves selected systemic metabolic alterations in ovariectomized diabetic female mice. These findings are relevant to experimental diabetes under estrogen-deficient conditions; however, they should be interpreted within the limitations of the OVX/alloxan model, which does not fully reproduce the multifactorial pathophysiology of postmenopausal type 2 diabetes. Therefore, the present results should be considered as evidence derived from an experimental hypoestrogenic diabetic model. Future studies are needed to define the underlying mechanisms, tissue-level effects, dose–response profile, oral efficacy, long-term safety, and translational potential of MOR.

## Data Availability

The original contributions presented in this study are included in the article. Further inquiries can be directed to the corresponding authors.

## Abbreviations

The following abbreviations are used in this manuscript:

ALT	Alanine aminotransferase
ANOVA	Analysis of variance
AST	Aspartate aminotransferase
AUC	Area under the curve
BUN	Blood urea nitrogen
CMC	Carboxymethylcellulose
DIAB	Diabetes
GLI	Glibenclamide
i.p.	Intraperitoneal
MOR	Morin
OGTT	Oral glucose tolerance test
OVX	Ovariectomy/ovariectomized
SEM	Standard error of the mean
SHAM	Sham-operated
VEH	Vehicle
